# Game-Based Meditation Therapy to Improve Posttraumatic Stress and Neurobiological Stress Systems in Traumatized Adolescents: Protocol for a Randomized Controlled Trial

**DOI:** 10.2196/19881

**Published:** 2020-09-23

**Authors:** Angela A T Schuurmans, Karin S Nijhof, Ron Scholte, Arne Popma, Roy Otten

**Affiliations:** 1 Behavioural Science Institute Radboud University Nijmegen Netherlands; 2 Department of Research and Development Pluryn Nijmegen Netherlands; 3 Praktikon Nijmegen Netherlands; 4 Tranzo Tilburg University Tilburg Netherlands; 5 Department of Child and Adolescent Psychiatry Vrije Universiteit medisch centrum De Bascule Amsterdam Netherlands; 6 Research and Education Advancing Children’s Health Institute Department of Psychology Arizona State University Tempe, AZ United States

**Keywords:** Adolescents, Autonomic nervous system, Cortisol, Meditation, Neurofeedback, Posttraumatic stress, Randomized controlled trial, Trauma

## Abstract

**Background:**

Many adolescents in residential care have been exposed to prolonged traumatic experiences such as violence, neglect, or abuse. Consequently, they suffer from posttraumatic stress. This not only negatively affects psychological and behavioral outcomes (eg, increased anxiety, depression, and aggression) but also has adverse effects on physiological outcomes, in particular on their neurobiological stress systems. Although current evidence-based treatment options are effective, they have their limitations. An alternative to traditional trauma treatment is meditation-based treatment that focuses on stress regulation and relaxation. Muse is a game-based meditation intervention that makes use of adolescents’ intrinsic motivation. The neurofeedback element reinforces relaxation abilities.

**Objective:**

This paper describes the protocol for a randomized controlled trial in which the goal is to examine the effectiveness of Muse (InteraXon Inc) in reducing posttraumatic stress and normalizing neurobiological stress systems in a sample of traumatized adolescents in residential care.

**Methods:**

This will be a multicenter, multi-informant, and multimethod randomized controlled trial. Participants will be adolescents (N=80), aged 10 to 18 years, with clinical levels of posttraumatic symptoms, who are randomized to receive either the Muse therapy sessions and treatment as usual (intervention) or treatment as usual alone (control). Data will be collected at 3 measurement instances: pretest (T1), posttest (T2), and at 2-month follow-up. Primary outcomes will be posttraumatic symptoms (self-report and mentor report) and stress (self-report) at posttest. Secondary outcomes will be neurobiological stress parameters under both resting and acute stress conditions, and anxiety, depression, and aggression at posttest. Secondary outcomes also include all measures at 2-month follow-up: posttraumatic symptoms, stress, anxiety, depression aggression, and neurobiological resting parameters.

**Results:**

The medical-ethical committee Arnhem-Nijmegen (NL58674.091.16) approved the trial on November 15, 2017. The study was registered on December 2, 2017. Participant enrollment started in January 2018, and the results of the study are expected to be published in spring or summer 2021.

**Conclusions:**

Study results will demonstrate whether game-based meditation therapy improves posttraumatic stress and neurobiological stress systems, and whether it is more effective than treatment as usual alone for traumatized adolescents.

**Trial Registration:**

Netherlands Trial Register NL6689 (NTR6859); https://www.trialregister.nl/trial/6689

**International Registered Report Identifier (IRRID):**

DERR1-10.2196/19881

## Introduction

### Background

Rates of chronic traumatic exposure among adolescents in residential care are staggering. Over 90% of these adolescents have been exposed to domestic violence, neglect, or emotional, physical, or sexual abuse at a young age [1]. Most of these traumatic experiences take place in the primary caregiving environment, even though caregiver support is essential for adolescents’ attachment, resilience, and stress adaptation [2]. Abuse by caregivers is probably the most interfering stressor that they can experience. Early traumatization interferes with adolescents’ healthy development [3]. It can have devastating effects on their psychosocial development [2], physical health [4], and neurobiological stress response systems [3,5-7].

### Neurobiological Stress Systems

Stress activates the autonomic nervous system and the hypothalamic-pituitary-adrenal axis to produce an appropriate stress response. The autonomic nervous system consists of the sympathetic nervous system and the parasympathetic nervous system. Activation of the sympathetic nervous system occurs rapidly after stress exposure—usually within milliseconds to seconds. When confronted with a stressor, the sympathetic nervous system activates physiological processes frequently referred to as the *fight or flight* response [8]. The increase in sympathetic nervous system activity is reflected by a shorter pre-ejection period (the length of time between the contraction of the heart and the ejection of blood out of the heart into the aorta), corresponding with a higher heart rate [9]. When the stressor disappears, the parasympathetic nervous system inhibits sympathetic activation and facilitates bodily homeostasis (ie, recovery and digestion) [8]. Generally, it has been assumed that the sympathetic nervous system and parasympathetic nervous system are reciprocally coupled: when sympathetic nervous system activity increases, parasympathetic nervous system activity decreases, and vice versa. However, various individual variations of autonomic nervous system and parasympathetic nervous system contributions to stress responses exist [10,11]. One key parameter of the parasympathetic nervous system is respiratory sinus arrhythmia, also referred to as vagal tone. Respiratory sinus arrhythmia is a measure of heart rate variability that represents parasympathetic nervous system control over heart rate and occurs at the frequency of respiration and that facilitates adaptive responses to the environment [12,13]. Lower respiratory sinus arrhythmia is associated with more internalizing and externalizing problems [14] and may increase the risk on internalizing problems after trauma [15,16].

The response of the hypothalamic-pituitary-adrenal axis is slower and has a longer duration than that of the sympathetic nervous system [8,17]. The primary effectors of the hypothalamic-pituitary-adrenal axis system are glucocorticoids (steroid hormones). These have a slower onset and longer duration than the catecholamines of the sympathetic nervous system. Stress activates the paraventricular nucleus of the hypothalamus, which releases corticotropin-releasing hormone within a few minutes, usually peaking 15 minutes after the stressor [18]. This stimulates the production of adrenocorticotropic hormone by the pituitary, which in turn signals the adrenal gland to release cortisol. Cortisol is also produced during nonstressful situations, referred to as basal cortisol [17]. Research in adolescents on the impact of trauma on cortisol shows evidence for a link between trauma and hypothalamic-pituitary-adrenal axis alterations but is characterized by the lack of a clear physiological profile. Exposure to trauma has been related to increased [19,20] and decreased cortisol levels [19-21].

In healthy individuals, the autonomic nervous system and the hypothalamic-pituitary-adrenal axis together generate adaptive responses in the face of acute threat. However, the experience of multiple, prolonged traumatic events can result in chronic activation of these stress systems, even when the original stressor has disappeared [2]. The unpredictable and ongoing nature of the traumatic events make these adolescents feel as if they are under constant threat of survival [22]. Unsurprisingly, the adverse effects of trauma become more severe and pervasive as the trauma lasts for a long period or the number of traumatic events increases [1,23].

### Trauma Treatment

Although trauma-focused cognitive behavioral therapy and eye movement desensitization and reprocessing are well-established effective treatments for traumatized adolescents [24], there are some inherent limitations. Both treatment models incorporate exposure techniques, verbal expression, and rely on the integration of cognition, emotion, and physiology. In order for the therapy to succeed in promoting change, sufficient capacity to regulate these systems is required [25]. Yet, traumatized adolescents often lack the capacity to control and regulate their impulses and emotions [2,26], and even when adolescents do exhibit the necessary skills in order for therapy to succeed in promoting change, residential care provides an often chaotic living environment that impedes the development of a therapeutic relationship and treatment progress [27]. Also, adolescents are often not motivated to talk about their experiences and re-experience their traumatic past [28].

Given the difficulties faced in traditional trauma treatment, alternative forms of intervention have gained popularity. The focus has shifted from mainly cognitive-oriented and verbally dependent therapies to interventions that target physiological sensations. One promising approach is fostered by meditation interventions that focus on adolescents’ stress regulation abilities. Meditation techniques (eg, deep-breathing practices) focus on one’s own bodily sensations and increase a sense of control over the body. Not only can feelings of stress be reduced and emotion-regulation capacities improved, individuals also showed beneficial changes in cardiovascular activity [29-31]. Meditation can restore activity and connectivity in brain regions associated with posttraumatic symptoms [32], lead to more balanced patterns of neurobiological stress responses [33], and modulate both hypothalamic-pituitary-adrenal axis and autonomic nervous system reactivity [34]. Research on the effectiveness of meditation interventions for posttraumatic stress shows encouraging findings. Most studies have been conducted among adults [32-36], but promising outcomes have been reported in traumatized adolescents too [37].

### Game-Based Therapy

This study aims to test the efficacy of game-based neurofeedback meditation therapy as an addition to treatment as usual in a population of traumatized adolescents in residential care. Gaming forms a novel strategy to engage adolescents into treatment and holds several advantages over traditional therapy. Videogame or gamified interventions make use of adolescents’ intrinsic motivation [38], while conventional treatment often depends upon imparting psychoeducational information, a didactic style of learning that contains few elements that are intrinsically motivating. Yet, motivation is an important predictor of treatment effectiveness [39], and adolescents in residential institutions are usually characterized by a lack of motivation to change their behavior [40]. Gamified treatment teaches adolescents techniques and skills but with less thinking and more doing [38]. This way of learning suits them better than memorizing certain principles [41]—conventional therapy—does [42]. Game-play is characterized by its repetitive nature. Repetition promotes long-term learning [43], and thus may foster generalization of its effects to adolescents’ daily lives. There have been some studies [44-46] conducted among adolescents in residential institutions that evaluated biofeedback videogame interventions. Results showed high user satisfaction, minimal attrition, and improved emotion-regulation [44-46].

Recently, the authors conducted a feasibility study [47] that evaluated 3 game-based meditation interventions in traumatized adolescents in residential care. The interventions incorporated either bio- or neurofeedback and were assessed on their potential to improve physiological stress regulation, user satisfaction, and preliminary effectiveness on posttraumatic symptoms, stress, depression, and aggression. The intervention that was evaluated positive on all outcomes and considered the best fit was Muse (InteraXon Inc)—a game-based neurofeedback meditation intervention.

### Objectives

The primary aim of this study is to investigate the effectiveness of Muse as an addition to treatment as usual in reducing posttraumatic symptoms (self- and mentor report) and stress (self-report) compared with treatment as usual in traumatized adolescents in residential care. We expect that playing Muse will result in a greater reduction of posttraumatic symptoms and stress than treatment as usual alone.

As a secondary aim, we will investigate the effectiveness of Muse on neurobiological stress systems under both resting and social stress conditions. It is hypothesized that participants who play Muse will show normalized neurobiological parameter compared to participants in the control group. We will also assess the effects of playing Muse on anxiety (self-report), depression (self-report) and aggression (self- and mentor report). It was expected that playing Muse would result in reduced anxiety, depression, and aggression.

## Methods

### Study Design

This study is a multicenter randomized controlled trial with 2 parallel intervention groups. Outcomes will be compared at 3 measurement instances: before the intervention (T1), immediately after the intervention (T2), and at a 2-month follow-up. Participants will be randomly assigned to the intervention or the treatment-as-usual (control) group, stratified by gender and intellect to ensure equal ratios of participants in both groups.

### Study Setting

Recruitment will take place in 3 residential institutions in The Netherlands that provide open and secured care for children and adolescents with and without intellectual disability. Residential care is the most intensive form of youth care and includes out-of-home placement and 24-hour care. These adolescents are unable to live at home due to severe psychiatric or behavioral problems, parental problems, or an unsafe home environment—often a combination of all of these. Adolescents live in group homes with group care workers as substitute care givers and receive treatment to target problem behavior. Residential care is often seen as a last-resort solution when there are no other options for treatment [48].

### Inclusion and Exclusion Criteria

Inclusion criteria are (1) clinical levels of posttraumatic symptoms, measured as a score of 30 or higher on the Children’s Revised Impact of Event Scale (CRIES-13) [49]; (2) age between 10 and 18 years; (3) capable of understanding and speaking Dutch; (4) active informed assent to participate in the study from participants themselves and active consent from legal guardians when participants are under the age of 16 (obtained by the first author). Exclusion criteria are (1) negative clinician advice, for example, when the participant already has other forms of treatment and the clinical fears that treatment burden would become too heavy (at this stage, participants are not randomized yet, so this exclusion criterion will affect both groups equally); (2) simultaneous participation in another clinical intervention study; (3) acute psychotic symptoms; (4) current or recent (within the previous 3 months) trauma treatment, specifically eye movement desensitization and reprocessing or trauma-focused cognitive behavioral therapy specifically targeting posttraumatic symptoms. There are no restrictions for other types of interventions that participants may receive (eg, medication, individual therapy, or group therapy). We will keep track of additional interventions and use them as covariates in the analyses, if necessary.

### Recruitment

At adolescents’ admission to the residential institutions taking part in the study, the CRIES-13 is included in the standard questionnaire battery that adolescents fill out at intake. Adolescents with a clinical score of posttraumatic symptoms on the CRIES-13 (≥30), filled in less than 3 months before T1, will be considered eligible participants for this study. We will include adolescents with clinical levels of posttraumatic symptoms rather than adolescents who qualify for the diagnosis of posttraumatic stress disorder because many adolescents who do not meet the criteria for posttraumatic stress disorder do suffer from posttraumatic symptoms and are in need of treatment [50].

Additionally, clinicians will be asked whether the adolescent in question can be invited to take part in this study. After clinician consult, eligible participants will be contacted by the coordinating researcher who will explain the studies. We will obtain verbal and written assent from all participants, and when they are younger than the age of 16, also written consent from their legal guardians. Potential participants will be invited for an individual meeting with the coordinating researcher who will explain the study. Participants will be explicitly informed about the study design and that they can quit study participation at any moment without disadvantages. The researcher will also bring information letters with information on all aspects of the study. When adolescents initially agree to participate, they will be given the information letters, and 2 to 3 weeks later, they will be asked for their written assent and invited for the pretest measurement (T1).

### Allocation and Randomization

If all inclusion and no exclusion criteria are fulfilled, participants will be randomly assigned (1:1 ratio) to 1 of the 2 groups using a randomization schedule that is generated by a Python script and stratified by gender, intellect, and residential institution. Allocation to groups is not masked, but participants and their mentors will not be informed about the specific expectations regarding posttraumatic symptoms and neurobiological stress reactivity. We will explain that Muse is a relaxation app, designed to help participants deal with stress. Participant enrollment and assignment to the groups will be performed by the first author.

### Interventions

All participants receive treatment as usual: the treatment as recommended by their clinicians regardless of this study (eg, individual or family therapy; medication). There are no restrictions for the type of interventions that participants can receive other than trauma-focused treatment, we only keep track of them. Participants in the intervention group will receive the intervention sessions as an addition to treatment as usual.

Muse is a game-based meditation app that is played on an iPad with a electroencephalography (EEG)–based headband that utilizes real-time neurofeedback. The intervention takes 6 weeks and consists of two 15- to 20-minute gameplay sessions per week. If participants are unable to make it to a session, this session will be rescheduled. All participants will complete 12 gameplay sessions in total. These sessions will take place in an office room located on the campus of the residential institution. Muse includes 10 relaxation tutorials that resemble elements of trauma-focused cognitive behavioral therapy such as deep-breathing techniques [42]. The tutorials are followed by 3-minute meditation sessions. The meditation tutorials are in English, so we have created a standardized protocol with Dutch translations of each tutorial. The gameplay sessions will be supervised by research assistants who are trained to explain the tutorials according to this protocol.

Participants can choose the in-game environment that will be shown during the meditation sessions (eg, beach, rainforest, city park). Before each tutorial, a short calibration will take place, during which the headband records the participants’ brain activity during rest, as a reference point for the upcoming meditation session. The tutorials are followed by 3-minute meditation sessions. Participants will complete at least 2 tutorials and subsequent meditation sessions each intervention session. During the meditation sessions, participants hear sounds in the in-game environment that they have chosen. The EEG-based headband provides real-time neurofeedback that is reflected by the intensity of activity in the environment. When the participant's mind is calm, the environment shows calm and settled winds, but these winds will pick up and blow when the participant's mind becomes more active. When participants succeed in remaining in a calm state for a sufficient amount of time, they will hear birds whistling. The neurofeedback element of the intervention involves retraining brain patterns through operant conditioning. Neurofeedback reinforces individuals’ relaxation abilities [51] and can reduce posttraumatic symptoms [52].

After each meditation session, participants get feedback on their performance through a series of simple graphs. In order to motivate, Muse calculates points, provides awards, and sets goals and challenges. Performances are saved and tracked so participants can see their progress over time.

### Study Procedure

See Table 1 for an overview of all measurements and outcomes. The pretest (T1) will take place in week 1 and will include an interview, an aquatic video, a social stress task, and a hair cortisol measurement. During the interview, the self-report questionnaires will be administered. We will conduct interviews rather than let participants fill in the questionnaires themselves, to ensure cooperation and comprehension. The interview format is, in particular, suitable for adolescents with lower intelligence, since this makes it easier for them to ask for additional explanation when they do not understand the question. Adolescents in residential care are often school dropouts and some of them may have trouble with reading, while they can easily answer the questions when these are read out aloud. The interviews will take approximately 30 minutes, with breaks in-between, and will be conducted by the coordinating researcher.

**Table 1:** Overview of all measurement moments and outcomes.

Time point	Study timeline	Procedure	Measures
T0	≤3 months before T1	Screening	Questionnaire
			Posttraumatic stress (CRIES-13^a^)
T1	Week 1	Pretest measurement	Questionnaires
			Posttraumatic symptoms (CROPS^b^ and PROPS^c^)
			Stress, depression, anxiety (DASS-21^d^)
			Aggression (RPQ^e^ and PRPA^f^)
			Neurobiological activity during rest
			Basal ANS^g^ activity
			Basal HPA^h^ axis activity
			Neurobiological reactivity to acute stress
			ANS reactivity
			HPA axis reactivity
—	Week 2-7	Intervention	—
T2	Week 8	Posttest measurement	Questionnaires
			Posttraumatic symptoms (CROPS and PROPS)
			Stress, depression, anxiety (DASS-21)
			Aggression (RPQ and PRPA)
			Neurobiological activity during rest
			Basal ANS activity
			Basal HPA axis activity
			Neurobiological reactivity to acute stress
			ANS reactivity
			HPA axis reactivity
Follow-up	Week 16	Two-month follow-up	Questionnaires
			Posttraumatic symptoms (CROPS and PROPS)
			Stress, depression, anxiety (DASS-21)
			Aggression (RPQ and PRPA)
			Neurobiological activity during rest
			Basal ANS activity
			Basal HPA axis activity

^a^CRIES-13: Children’s Revised Impact of Event Scale.

^b^CROPS: Child Report of Posttraumatic Symptoms.

^c^DASS-21: Depression Anxiety Stress Scales.

^d^PROPS: Parent Report of Posttraumatic Symptoms.

^e^PRPA: Parent-rating scale for Reactive and Proactive Aggression.

^f^RPQ: Reactive and Proactive Aggression Questionnaire.

^g^ANS: autonomic nervous system.

^h^HPA: hypothalamic-pituitary-adrenal.

Participants will watch an aquatic video for 5 minutes [53] to derive basal autonomic nervous system parameters during rest, in order to compare to the autonomic nervous system parameters derived during the stress task. The social stress task is an adapted and combined version of the Trier Social Stress Task for Children (TRIER-C) [54,55] and the Sing-a-Song Stress Test (SSST) [56]. Participants receive the introduction of a story and are told that they have 5 minutes to compose the end of the story. Then, they will present their story for 4 minutes in front of a camera. Unlike the original TRIER-C, the judge panel will be replaced by a video camera [57]. Participants will be recorded, allegedly for later assessment by a group of peers who will judge their performance. If the participant does not complete the 4 minutes for presenting, the researcher can use a standard set of prompts to encourage further narration. Immediately after the 4 minutes, participants will be asked to fill in a 3-item manipulation check to rate how nervous the presentation task has made them on a scale from 1 (not nervous at all) to 10 (very nervous). Next, participants are given a booklet with song texts and are told that they have to sing a song in front of the camera. Participants have 30 seconds to choose a song and are expected to sing the song aloud for 30 seconds. Again, they will be asked to fill out the manipulation check immediately after the task. For an overview of the stress task, see Table 2. Participants will not be fully debriefed until they completed the second stress task at T2.

**Table 2:** Overview of the social stress task to measure neurobiological reactivity to acute stress.

Task timeline			Activity		ANS^a^ recordings		Cortisol samples
**Before**							
	–20 minutes	—		—		–20	
	–5 minutes	Aquatic video		Basal ANS parameters		—	
	0 minutes	—		—		Pre	
**Start**							
		Anticipation speech task		ANS reactivity		—	
		Speech task		ANS reactivity		—	
		Manipulation check		—		—	
		Anticipation song task		ANS reactivity		—	
		Song task		ANS reactivity		—	
		Manipulation check		—		—	
**After**							
	0 minutes	Recovery		ANS recovery		Post	
	10 minutes	—		—		+10	
	20 minutes	—		—		+20	
	40 minutes	—		—		+40	

^a^ANS: autonomic nervous system.

The posttest (T2) will take place in week 8 and includes, like the pretest (T1), an interview, aquatic video, social stress task, and a hair cortisol measurement. Since participants will be exposed to the social stress task twice, it is expected that they show habituation-related decreased levels of stress at T2 [58,59]. To lower the risk for stress habituation and to improve methodological rigor for the second social stress task, participants will be given another story introduction that is comparable to the story that will be used for T1. At the end of T2, participants will be debriefed and told that the video recordings were deleted immediately after the measurement session.

The 2-month follow-up will take place in week 16 and includes an interview, aquatic video, and a hair cortisol measurement. The 2-month follow-up measurement is conducted to provide additional information about potential long-term intervention effects. Therefore, we will use questionnaires and the parameters to measure neurobiological activity during rest that are relatively simple to obtain (ie, a 5-minute autonomic nervous system measurement during the aquatic video and cutting the hair). Measuring neurobiological reactivity requires the execution of a stress task that is time-consuming, and even more important, eliciting stress among traumatized adolescents is not without risks and should only be done when essential for research purposes. For those reasons, neurobiological reactivity to acute stress was not included at 2-month follow-up measurement.

Participants will receive a gift voucher of €15 (approximately US $17.86) at T1, a gift voucher of €10 (approximately US $11.91) and a stress ball at T2, and a gift voucher of €15 at 2-month follow-up measurement. Participants’ mentors (ie, the group home worker with whom they have the most contact) will fill in the questionnaires in the same week as when the measurements with the participants are conducted.

### Measures

#### Questionnaires

The screening instrument is the CRIES-13 [49], a questionnaire with 13 four-point items to screen whether adolescents suffer from posttraumatic symptoms.

Posttraumatic symptoms will be measured by participants’ self-report as well as by mentor report. The Child Report of Posttraumatic Symptoms (CROPS) [60] is a self-report questionnaire that consists of 24 three-point items. The Parent Report of Posttraumatic Symptoms (PROPS) [60] is a 30-item questionnaire that measures posttraumatic symptoms as reported by parents or other caretakers. In this study, participants’ group home mentors will fill out the questionnaire. Several studies have demonstrated the validity and reliability of the CROPS and the PROPS in general populations [61]. Additionally, the CROPS has been validated in a sample of juvenile offenders [62]. The CROPS and the PROPS have been jointly developed so that the CROPS focuses on internal thoughts and feelings, whereas the PROPS focuses on observable behaviors.

Stress will be examined with the 7-item stress subscale of the Depression Anxiety Stress Scales (DASS-21) [63,64]. The DASS-21 is a self-report questionnaire that in total consists of 21 four-point items. Its validity and reliability are good and the subscales have an excellent internal consistency [63].

Anxiety will be measured with the 7-item anxiety subscale of the DASS-21 [63,64].

Depression will be measured with the 7-item depression subscale of the DASS-21 [63,64].

Aggression will be measured by participants self-report as well as by mentor report. The Reactive and Proactive Aggression Questionnaire (RPQ) [65] is a self-report questionnaire that is composed of 23 three-point items. The RPQ consists of proactive aggression and reactive aggression subscales. Its validity and reliability are good, and both subscales have good internal consistency [65]. The Parent-rating scale for Reactive and Proactive Aggression (PRPA) [66] is a questionnaire that can be filled out by parents or other caretakers. The PRPA has 11 three-point items and consists of reactive aggression and proactive aggression subscales. The PRPA total score and both its subscale scores have good validity [66].

#### Neurobiological Activity During Rest

Autonomic nervous system parameters will be measured with the *Vrije Universiteit* Ambulatory Monitoring System (VU-AMS) [67,68]. This a lightweight ambulatory device that records electrocardiograms and changes in thorax impedance with 5 electrodes that are placed on participants’ chest and 2 that are placed on the back. The electrocardiogram has a sampling rate of 1000 Hz and heart rate is obtained from the time between 2 adjacent R peaks. Heart rate data will be extracted and visually inspected for artifacts with the *Vrije Universiteit* Data Analysis and Management Software program (VU-DAMS; version 4.0) [69]. We will derive heart rate, respiratory sinus arrhythmia, pre-ejection period, respiration rate, and skin conductance parameters from the VU-AMS recordings.

Basal autonomic nervous system parameters during rest will be derived from VU-AMS recordings while participants are watching an aquatic video for 5 minutes [53].

For basal hypothalamic-pituitary-adrenal axis activity, basal cortisol levels will be measured in participants’ hair. Whereas saliva captures real-time cortisol levels that are subject to major fluctuations [70], hair cortisol provide a reliable way to assess average long-term activity of the hypothalamic-pituitary-adrenal axis (thus cortisol exposure over longer time). Hair samples will be cut as close to the scalp as possible from a posterior vertex position. At least 15 mg of the most proximal 1.5 cm of each hair sample will be used for analysis—representing the basal level over the 6 weeks before the hair sample was taken. Hair processing and analyses will be conducted by the Laboratory of Endocrinology of the Erasmus Medical Center, Rotterdam, the Netherlands. Hair samples will be washed in isopropanol and after solid phase extraction, hair cortisol will be quantified by liquid chromatography–tandem mass spectrometry [71].

#### Neurobiological Reactivity to Acute Stress

For autonomic nervous system reactivity to acute stress, autonomic nervous system parameters will be measured while participants complete the social stress task (see Table 2). We will derive autonomic nervous system parameters during the following segments of the stress task: (1) anticipation speech task, (2) performing speech task, (3) anticipation song task, (4) song task, and (5) recovery.

For hypothalamic-pituitary-adrenal axis reactivity to acute stress, salivary cortisol levels will be measures before and after completion of the social stress task (see Table 2). Six saliva samples will be obtained with a collection device (Salivette; Sarstedt AG and Co) twenty minutes (–20), immediately before the social stress task (pre), immediately after (post), ten minutes (+10), twenty minutes (+20), and forty (+40) minutes after the task (Table 2). Cortisol levels will be measured using the liquid chromatography–tandem mass spectrometry method with CHS MSMS Steroid kit (PerkinElmer).

### Sample Size Calculation

Power calculations were performed using G*Power [72]. An a priori power analysis for analysis of variance (repeated measures, within-between interaction) was conducted. Based on previous meditation-based interventions for posttraumatic stress [32,34-36,73], we expected a small to medium effect size of *d*=0.30 and a repeated measures correlation of 0.60. To achieve a statistical power of at least .80, a sample size of 72 participants was required. We aim to include 80 participants in total to allow for 10% attrition, which was estimated from previous studies [44,45,47] on game-based interventions in this population.

### Statistical Analysis

To assess intervention efficacy, we will use repeated measure analysis of variance to detect differences in mean outcome scores between the 2 groups at T2 and at 2-month follow-up. All analyses will be conducted in accordance with the intention-to-treat principle. Missing data will be imputed using Markov Chain Monte Carlo [74]. Results will be expressed as differences in mean scores between the 2 groups with 95% confidence intervals. *P* values<.05 will be considered statistically significant.

### Ethical and Safety Issues

Ethical review and approval of this study has been provided by the medical-ethical committee Arnhem-Nijmegen (protocol NL58674.091.16). All substantial amendments will be presented to the committee and competent authority. Before participants are included in the study, they will be informed about the study design, including randomization. Participants who are assigned to the control group will be offered the opportunity to play Muse after the study has ended. Participants can withdraw from participation at any time without consequences.

Participant privacy will be protected by allocating identification numbers to personal information that is traceable in a separate file using double-key encryption. Data will be analyzed in a way that no conclusions can be drawn about individual participants. The 3 researchers that will have access to the data work at the residential institutions where the study is being conducted. Data entry will be double-checked to ensure accuracy. Biological materials will be stored in a locked cabinet or a locked medical freezer at Pluryn until the end of the study. The material will be destroyed after laboratory analysis, as requested by the medical-ethical committee that approved the study.

Adverse events reported by participants or group care workers or observed by the researchers will be recorded and assessed in collaboration with participant’s clinician. The potential relation to the intervention will be examined and participants will be followed until they are reached stable. The risk in participating for this study is considered negligible, but if a participant seems negatively affected by the measurements or intervention, study participation will be discussed with the clinician and discontinued, if necessary.

## Results

This study has been registered (Netherlands Trial Register NL6689 [NTR6859]). This study was approved by the medical-ethical committee Arnhem-Nijmegen (NL58674.091.16) on November 15, 2017. Participant enrollment started in January 2018. The results of the study will be published in international journals and presented at international conferences. Research findings are expected to be published in the spring or summer of 2021.

## Discussion

This study is the first to examine the effectiveness of a game-based meditation intervention in a clinical population of traumatized adolescents in residential care. To test the effectiveness of interventions within this population is crucial, since early alterations of these systems increase the risk of psychopathology at a later age [75]. At an early age, neurobiological stress systems contain high rates of plasticity [76], so adolescents could benefit substantially from effective treatment.

A particular strength of this study is that we use a multimodal, multi-informant approach with different types of assessment (ie, questionnaires and physiological measures) and different informants (ie, self-report and mentor-report). This type of approach is the most accurate way to assess and monitor adolescent mental health [77]. Additionally, we will measure hypothalamic-pituitary-adrenal axis and autonomic nervous system reactivity, since both neurobiological stress systems are hypothesized to play a role in the development and maintenance of posttraumatic symptoms [3]. To gain understanding in how trauma affects neurobiology, multisystem approaches need to be considered [78].

Limitations to the design of this study are the nonactive control group, and because of that, the lack of group concealment. Active control groups are more rigorous and leave no room for alternative explanations regarding attention, motivation, and behavioral expectation, but only when participants in both groups have the same expectations of improvement [79]. An optimal control group would require a game-based intervention that is comparable to that of Muse, but without its hypothesized working mechanisms. This is not feasible since this study will be conducted in residential institutions that have tight restrictions for casual gameplay. Clinicians do not agree on implementing a gameplay intervention that is not expected to be beneficial for participants. The primary purpose of this study, however, is to test the effectiveness of Muse as an addition to treatment as usual, not to determine its superiority to another form of treatment. Therefore, treatment as usual can be considered as a valid control group [80]. Due to the inactive control group, masking of groups is not possible. Participants and mentors will be aware of the groups to which the participants are assigned. Thus, it might be possible that differential expectations will bias study outcomes.

## Abbreviations

CRIES-13: Children’s Revised Impact of Event Scale
CROPS: Child Report of Posttraumatic Symptoms
DASS-21: Depression Anxiety Stress Scales
PROPS: Parent Report of Posttraumatic Symptoms
PRPA: Parent-rating scale for Reactive and Proactive Aggression
RPQ: Reactive and Proactive Aggression Questionnaire
SSST: Sing-a-Song Stress Test
TRIER-C: Trier Social Stress Task for Children
VU-AMS: Vrije Universiteit Ambulatory Monitoring System
VU-DAMS: Vrije Universiteit Data Analysis and Management Software
